# Monitoring of miR-181a-5p and miR-155-5p Plasmatic Expression as Prognostic Biomarkers for Acute and Subclinical Rejection in *de novo* Adult Liver Transplant Recipients

**DOI:** 10.3389/fimmu.2019.00873

**Published:** 2019-04-24

**Authors:** Olga Millán, Pablo Ruiz, Lara Orts, Paula Ferré, Gonzalo Crespo, Miguel Santana, Virginia Fortuna, Luís Quintairos, Miguel Navasa, Mercè Brunet

**Affiliations:** ^1^Biomedical Research Center in Hepatic and Digestive Diseases (CIBERehd), Instituto de Salud Carlos III, Madrid, Spain; ^2^Pharmacology and Toxicology, Biochemistry and Molecular Genetics, Biomedical Diagnostic Center (CDB), IDIBAPS, Hospital Clinic of Barcelona, University of Barcelona, Barcelona, Spain; ^3^Liver Unit, IDIBAPS, Hospital Clinic of Barcelona, University of Barcelona, Barcelona, Spain

**Keywords:** biomarkers, miR-181a-5p, miR-155-5p, liver transplantation, TCMAR, SCR

## Abstract

**Background and Aims:** News strategies for the accurate assessment of the state of immunosuppression (IS) in liver transplant recipients are needed to prevent rejection and minimize drug-related side effects. miRNAs can potentially be used as diagnostic or prognostic biomarkers in transplant patients. This study evaluated the capacity of a plasmatic miRNA panel (miR-155-5p, miR-122-5p, miR-181a-5p, and miR148-3p) as an early non-invasive prognostic and diagnostic biomarker for T cell-mediated acute rejection (TCMAR) and subclinical rejection (SCR) in adult liver recipients.

**Methods:** A total of 145 liver recipients were included. All patients received a calcineurin inhibitor with or without mycophenolate mofetil and methylprednisolone. Plasmatic miRNA expression was assessed by qPCR before and at different time-points after liver transplantation.

**Results:** Seventeen patients experienced TCMAR, and eight were diagnosed with SCR during the protocol biopsy at the 3rd month post-transplantation. Pre-transplantation, miR-155-5p expression was significantly higher in TCMAR patients and in SCR patients than in non-rejectors, and miR-181a-5p expression was also significantly higher in SCR patients than in non-rejectors. Post-transplantation, before transaminase-level modification, significantly increased miR-181a-5p, miR-155-5p, and miR-122-5p expression was observed in TCMAR and SCR patients. Binary logistic regression analyses showed, post-transplantation, that TCMAR risk was better predicted by individual expression of miR-181a-5p (LOGIT = −6.35 + 3.87^*^miR-181a-5p), and SCR risk was better predicted by the combination of miR-181a-5p and miR-155-5p expression (LOGIT = −5.18 + 2.27^*^miR-181a-5p+1.74^*^miR-155-5p).

**Conclusions:** Pre-transplantation plasmatic miR-155-5p expression may be useful for stratifying low-immunologic-risk patients, and post-transplantation miR-181a-5p and miR-155-5p may be candidates for inclusion in early, non-invasive prognostic biomarker panels to prevent TCMAR or SCR and better identify patient candidates for IS minimization. Large prospective randomized multicenter trials are needed to refine the cut-off values and algorithms and validate the clinical usefulness of these biomarkers.

## Introduction

Despite great advances in immunosuppressive (IS) therapy, T cell-mediated rejection (TCMAR), which is the main form of rejection in liver transplantation (LT), has an incidence estimated ~21–27% (1, 2). Moreover, the diagnosis of TCMAR is based on the findings of liver biopsy, which is an invasive procedure that can result in certain complications. On the other hand, the side effects of IS therapy, such as increased risk of renal failure and neoplasia or the impairment of cardiovascular function, has led to strategies for IS reduction to minimize these deleterious events. Accurate methods for stratifying the net state of immune suppression are lacking. The availability of non-invasive biomarkers of an alloimmune response may provide physicians useful information to better identify patients at high risk of rejection and those who are good candidates for IS minimization post-transplantation and to achieve more personalized IS therapy. Monitoring these biomarkers may decrease the requirement for the use of biopsies (3). Furthermore, subclinical inflammatory lesions in the graft, such as portal inflammation with variable grades of fibrosis as described by Londoño et al. or histological signs of rejection without biochemical abnormalities (subclinical rejection; SCR), are less evaluated entities with uncertain significance and prognosis (4–6).

Several studies have shown the potential role of miRNA analysis as a non-invasive prognostic and diagnostic biomarker of TCMAR (7). The control of gene expression by miRNAs regulates many cellular functions, including differentiation, proliferation, cellular development, and functional regulation of the immune system, and miRNAs also participate in the cellular mechanisms of inflammatory responses (8, 9). There is evidence that miRNAs play an important role in post-transplant clinical events, including rejection, disease recurrence, and the development of tumors.

Publications on miRNAs in transplantation mostly focus on the field of kidney transplantation (10–12). In LT, emerging studies are evaluating the role of miRNA analysis as biomarkers for liver injury. Hepatocyte-derived miRNAs, such as miR-122, miR-148a, and miR-194, correlate with injury and acute rejection (AR). Serum levels of these miRNAs are increased in patients with liver injury and positively correlate with aminotransferase levels, but this increase occurs before aminotransferase levels are modified during AR, showing the prognostic capacity of these biomarkers (13). In fact, miR-122-5p is the most abundant liver-derived miRNA, constituting 70% of the total miRNA in the liver (14), and thus, a significant increase in this miRNA could be associated with hepatocyte damage, toxicity, or viral infection (15). Schmuck et al. also demonstrated that the levels of miR-122-5p, miR-133a, miR-148a-3p, and miR-194 are significantly higher in the bile of liver recipients who develop AR within the first 6 months after transplantation and during the AR episode (16). Recently, Shaked et al., by using the framework of the Immune Tolerance Network Immunosuppression Withdrawal (ITN030ST) and Clinical Trials in Organ Transplantation (CTOT-03) studies, identified in plasma two miRNAs (miR-483-3p and miR-885-5p) that, when combined together in a signature, could diagnose and predict liver rejection with high accuracy [area under the curve (AUC) = 89.5%; 95% CI = 82–96%; sensitivity = 83.8%; specificity = 87.1%; positive predictive value (PPV) = 72% and negative predictive value (NPV) = 93%] (7).

Local inflammation associated with rejection is tightly regulated by the different T helper (Th) subsets, such as Th1, Th2, and Th17. The differentiation of CD4+T cells into these different subsets is strongly influenced by cytokine signaling and by the expression of subset-specific transcription factors, such as miRNAs. Recently, it has been described that miR-155-5p controls the differentiation of CD4+T cells into Th cells (17, 18) and participates in the development of regulatory T cells (19). It has been reported that miR-155-5p can also act as a regulator of IFN-γ production in human T and NK cells (17, 20). Previous results from multicenter studies have shown that, in peripheral blood, intracellular T cell (CD4^+^ and CD8^+^ T cells) expression of IFN-γ correlates with graft rejection and clinical outcome and could be a useful prognostic and diagnostic biomarker in kidney and liver transplantation (21, 22). Several groups have demonstrated that miR-155-5p is overexpressed in renal allografts during acute cellular rejection (10, 12). Recently, Blaya et al. (23) showed that miR-155-5p expression is altered in both liver tissue and circulating inflammatory cells (peripheral blood mononuclear cells) during liver injury, thus regulating inflammatory cell recruitment and liver damage. Another miRNA closely related to the function of T lymphocytes is miR-181a. Li et al. demonstrated that T cell receptor (TCR) sensitivity and signaling strength can be modulated at the post-transcriptional level by miR-181a. The recognition of donor antigens by recipient T cells in secondary lymphoid organs initiates the adaptive inflammatory immune response leading to the rejection of allogeneic transplants. Allospecific T cells become activated through the interaction of their TCRs with an intact allogeneic major histocompatibility complex; the modulation of selection also argues that this miRNA might directly impact the mature T cell repertoire, which might further affect the onset and/or progression of the T cell alloresponse. miR-181a represents a novel class of regulatory molecules that can modulate the T cell response. Therefore, changes in its expression can regulate the alloresponse against the implanted graft and, consequently, may play a role in the development of rejection (24, 25). No data have been reported regarding the potential utility of evaluating plasmatic miRNA expression for predicting the risk of SCR in LT recipients.

The aim of this study was to evaluate the capacity of a plasmatic miRNA panel (miR-155-5p, miR-122-5p, miR-181a-5p, and miR148-3p) as an early non-invasive prognostic and diagnostic biomarker for TCMAR and (for the first time) for SCR in adult LT recipients.

## Methods

### Study Design and Patients

We conducted a prospective, observational study in a cohort of 145 adult patients undergoing LT in our center (Hospital Clínic Barcelona). Combined liver-kidney recipients were excluded. Only patients followed for at least 3 months after LT with plasma samples collected were considered. The study was approved by the Ethics Committees of the center, and all patients provided their written informed consent.

#### Patient Follow Up and Immunosuppressant Regimens

All LT recipients were managed by transplant hepatologists according to standardized protocols throughout the follow up. After discharge after transplantation, the patients visited the outpatient clinic monthly for the first 3 months and every 2 months thereafter during the first year. Clinical, demographical and laboratory data of the patients were collected. IS regimens were defined by the pre-transplant liver status according to the Child-Pugh classification. Child-Pugh A patients were given a double IS therapy consisting of tacrolimus (TAC) or cyclosporine (CsA) with target trough levels of 8–10 or 150–300 ng/ml, respectively, plus a tapering dose of corticosteroids to be withdrawn at 6 months after LT. Child-Pugh B and C patients, those transplanted due to acute liver failure and retransplanted patients who received induction therapy with a single dose of basiliximab immediately after LT, delayed the start (5th day) of TAC or CsA with target trough levels of 5–8 or 75–150 ng/ml, respectively, mycophenolate mofetil (MMF) 2,000 mg daily, and a tapering dose of corticosteroids to be withdrawn at 6 months after LT. A low dose of corticosteroids (prednisone 2.5–5 mg) was maintained long-term in those patients transplanted due to an autoimmune disease.

#### Liver Biopsies and Rejection Episodes

Liver biopsies were performed if clinically indicated and if the following criteria were met: aspartate aminotransferase, alanine aminotransferase, alkaline phosphatase or direct bilirubin serum levels higher than 2-fold the upper limit of normal and absence of pathological findings in the abdominal ultrasound examination that could explain these biochemical abnormalities. All rejection episodes were biopsy proven, and severity was defined by the Rejection Activity Index (RAI) Score (26, 27). Moderate (RAI 5–6) and severe (RAI > 6) episodes were treated with steroid boluses (500 mg of methylprednisolone for three consecutive days), while mild episodes (RAI 3–4) were treated by increasing the dose and levels of current immunosuppressants. In addition, a per-protocol biopsy was performed at month 3 after LT in all patients in whom a clinically indicated biopsy had not been performed, unless there was a clinical contraindication (mainly, biliary complications). All these patients submitted to a per-protocol biopsy and had normal liver function tests and abdominal ultrasound examination. If histological signs of TCMAR were found, patients were diagnosed with subclinical rejection (SCR). No additional treatment was given for this event, but IS doses were maintained without minimization for at least 4 weeks after biopsy.

### Pharmacokinetic Monitoring

Therapeutic drug monitoring of CNi was performed in the Laboratory of Pharmacology. For each CNi, the trough concentration at the 1st week, on the 15th day, and at the 1st, 2nd, 3rd, 6th, 9th, and 12th month post-transplantation were analyzed. Whole blood TAC concentrations were determined by Tacrolimus-CMIA-Architect from Abbot (Wiesbaden, Germany), and whole blood CsA concentrations were measured by Cyclosporin-ACMIA-Dimension from Siemens (Siemens Healthcare Diagnostic, Deerfield, IL, USA) following the manufacturer's instructions. Fresh samples, without having been previously frozen, were analyzed daily. LGC Standard Proficiency Testing was ensured by the participation of our laboratory in the United Kingdom External Analytical Quality Assessment Service.

### Plasma miRNA Analysis

At the same time as the clinical visits and pharmacokinetic profiles, plasma miR-155-5p, miR-122-5p, miR-181a-5p, and miR-148-3p expression was assessed by quantitative real-time PCR (qPCR). We selected these miRNAs based on the results of previous studies that showed them as promising biomarkers in the diagnosis and prognosis of rejection and for their involvement in the response mechanisms that the immune system performs against the graft. A total of 4,785 determinations were performed: 1,305 determinations for each miR evaluated (145 patients, 9 visits, and 3 miRs), except for miR-148-3p, which had 870 determinations, because the follow up was performed until 3 months post-transplantation (145 patients, 6 visits, 1 miR). Blood samples (3 ml) were collected into EDTA-K3 tubes at the pre-transplantation visit and at each post-transplantation visit according to the study design. Blood samples were obtained prior to the immunosuppressant administration (pre-dose); at those points concurrent with rejection episodes, the samples were collected before any treatment change was made. After centrifugation (within 2 h) at 3,000 rpm for 10 min, plasma was collected and stored in RNase-free tubes at −70°C for batched analysis.

Total RNA was purified from 200 μL of patient plasma according to the manufacturer's instructions [miRCURY™ RNA Isolation Kits – Biofluids ref. #300112 from EXIQON (Denmark)]. Briefly, plasma components were lysed with the provided Lysis Solution, and proteins were precipitated with the provided Protein Precipitation Solution. Isopropanol was added to the collected supernatant, and the solution was loaded into the column. The solution was washed with Wash Solutions 1 and 2, the RNA was eluted with RNase-free water, and the concentration and quantity of the total RNA were measured at 260 nm and 280 nm (A260/A280) using a NanoDrop device (NanoDrop Technologies). Hemolysis contamination was tested using a spectrophotometer and measuring the oxyhemoglobin absorbance at 414 nm. An OD scan was performed from ~200 to 700 nm, and distinguishing absorbance peaks at 414 nm were used to disqualify hemolysis samples. Total RNA was reverse transcribed into cDNA following the manufacturer's instructions (miRCURY LNA™ Universal RT ref #203301 from EXIQON). cDNA served as a template for miRNA RT-qPCR amplification with locked nucleic acid (LNA) primers and SYBR Green master mix. The following specific LNA PCR primer sets, all from EXIQON (Denmark), were used: hsa-miR-155-5p LNA™ PCR primer set, UniRT (ref.# 204308); hsa-miR-181a- 5p LNA™ PCR primer set, UniRT (ref. # 206081); hsa-miR-122- 5p LNA™ PCR primer set, UniRT (ref, # 205664); hsa-miR-148a-3p LNA™ PCR primer set, UniRT (ref.# 205867); hsa-miR-103a-3p, LNA™ PCR primer set, UniRT (ref. 204063); and hsa-miR-191-5p, LNA™ PCR primer set, UniRT (ref. # 204306). To monitor the cDNA synthesis reaction, the synthetic spike-in UniSP6 was used for signs of inhibition (prior to the reverse transcription reaction, we added 1 μl of synthetic spike-in (108 copies/μl) per 20 ng sample RNA). PCRs were performed using a Light Cycler 480 instrument. The amplification profile was denatured at 95°C for 10 min followed by 45 amplification cycles of 95°C for 10 s and 60°C for 1 min. At the end of the PCR cycles, melting curve analyses were performed. Negative controls with 1 μg of MS2 carrier RNA as a mock template from the reverse transcription reaction were produced and profiled similarly to the samples. The amplification curves were analyzed using Roche LC Software for determination of Cq by the second derivative method. Average Cq values were normalized to the stably expressed reference miR-103a-3p and miR-191-5p, following the manufacturer's instructions. First, the Cq values for all samples were determined; the average Cq of miR-191-5p + miR-103-3p was calculated, and the ΔCq was calculated as the difference in Cq values between the miRNA target and the reference control (Cq average of miR-191-5p+miR-103-5p). Relative expression levels of target miRNAs were then evaluated within a sample according to the formula 2∧(-ΔCq), where high values corresponded to higher expression.

### Statistical Analyses

Demographic data and results of the molecular analyses were collected in a unified database. Samples were adjusted to a non-parametric distribution. Statistical differences between groups were assessed with the Mann–Whitney test and Kruskal–Wallis test, and correlations between miRNA expression and clinical events were assessed with Spearman's rho test. We used a receiver operating characteristic (ROC) curve analysis to define the optimal cut-offs for differentiating between patient groups with and without TCMAR or SCR. Optimal biomarker cut-off points to discriminate between patients with and without TCMAR were based on ROC curves and calculated with the best Youden index (28) (sensitivity + specificity−1). For the analysis of SCR, we treated the data of this group as an independent group during the evaluation period. Discriminatory capacity was defined by the AUC (0.7–0.8, acceptable; 0.8–0.9, excellent; >0.9, outstanding), with its 95% confidence interval (CI). All analyses were performed using SPSS 23.0 software (SPSS, Inc., Chicago, IL, USA). All data are presented as the median ± standard deviation (SD). A value of *P* ≤ 0.05 was considered statistically significant. To better evaluate not only the diagnostic capacity of the biomarkers evaluated in this study but also their prognostic utility, we included in the TCMAR box-plot data from patients who exhibited rejection at this time plus the pre-TCMAR data of the patients who had not yet exhibited rejection at this time but who would do so in a later profile. We did not consider data from rejector patients once the TCMAR episode was resolved in the TCMAR box-plot graph. A binary logistic regression model (26) was performed using NONMEM software [version 7.4.1; Icon development Solutions, Ellicott City, MD, USA; (29)] with the Laplacian first order conditional estimation method. TCMAR and SCR occurrence were evaluated as binary data and used as response variables (RVs), with 0 indicating no event, and 1 indicating occurrence of the event. As explanatory variables, miR-155-5p and miR-181a-5p plasmatic expression were used. The probability of the observed score was linked to explanatory variables through the logit transformation to ensure that the estimated probability fell between 0 and 1. Graphic evaluation of the output was performed with R software (30). As a model evaluation, a visual predictive check (vpc) after 1,000 simulations using vpc R package (31) and a bootstrap analysis after 1,000 resamplings using Perl Speaks NONMEM (PSN) were performed (32, 33).

## Results

### Study Patients

From September 2014 to July 2018, 178 patients were included. Twelve patients remained on the LT waiting list at the end of the inclusion period, 6 died before undergoing LT, and 15 patients did not met the minimum follow-up period for several reasons: 5 died before month 3; 1 transplant could not be performed because of a technical impossibility found during surgery; 4 patients withdrew consent; and 5 had no complications but a shorter than 3-month follow up at the time of analysis. The final study cohort consisted of 145 individuals. The main characteristics are shown in Table 1. Most patients were males (72.4%), with a mean age of 56.5 years. The main etiologies of primary liver disease were HCV and alcohol, and hepatocellular carcinoma was the indication for LT in 47.6% of patients. The majority of donors were donors after brain death, with a median age of 58.5 years. Regarding the immunosuppressive regimen, 79.3% of patients received TAC (with or without MMF), while the remaining 20.7% had cyclosporine A. Among patients with HCV as primary disease (*n* = 53), 8 of them were positive for HCV RNA at the time of transplant. As expected, all of them had HCV recurrence after LT.

**Table 1:** Characteristics of 145 liver transplant recipients.

		**Liver transplant recipients** ***N*** **=** **145**				
		**Total**	**Non-rejectors. *N* = 120**	**Rejectors *N* = 17**	**Subclinical rejectors *N* = 8**	***P*-value**
Sex (male)		105 (72.4%)	89 (74.2%)	12 (70.6%)	4 (50%)	0.35
Recipient age (years)		56.5 ± 8.6	58.5 ± 8.5	51.0 ± 9.6	57.0 ± 7.9	0.74
Primary disease	Alcohol	34 (23.5%)	27 (22.5%)	5 (29.4%)	2 (25%)	0.50
	HCV	44 (30.3%)	38 (31.7%)	4 (23.5)	2 (25%)	
	HCV + Alcohol	9 (6.2%)	8 (6.7%)	1 (5.9%)	0	
	HBV	10 (6.9%)	10 (8.3%)	0	0	
	Autoimmune	6 (4.1%)	4 (3.3%)	0	2 (25%)	
	PBC	10 (6.9%)	7 (5.8%)	2 (11.8%)	1 (12.5%)	
	PSC	4 (2.8%)	3 (2.5%)	1 (5.9%)	0	
	Cryptogenic	7 (4.8%)	5 (4.2%)	1 (5.9%)	1 (12.5%)	
	NASH	11 (7.6%)	9 (7.5%)	2 (11.8%)	0	
	Other	10 (6.9%)	9 (7.5%)	1 (5.9%)	0	
Hepatocellular carcinoma		69 (47.6%)	58 (47.9%)	7 (42.8%)	4 (50%)	0.94
Donor age (years)		58.5 ± 14.4	58.6 ± 15.5	53.0 ± 15.3	68.5 ± 8.4	0.22
Cold ischemia time (minutes)		460.6 ± 146	443.5 ± 133.8	430 ± 112.2	515.0 ± 133.8	0.65
Type of donor	Living donor	1 (0.7%)	1 (0.8%)	0	0	0.61
	DBD	123 (84.8%)	103 (85.8%)	14 (82.4%)	6 (75%)	
	DCD	21 (14.5%)	16 (13.4%)	3 (17.6%)	2 (25%)	
Immunosuppressive regimen	TAC + PDN	61 (42.1%)	49 (40.8%)	8 (47.1%)	4 (50%)	0.45
	TAC + MMF + PDN	54 (37.2%)	43 (35.8%)	8 (47.1%)	3 (37.5%)	
	CsA + PDN	15 (10.3%)	14 (11.7%)	0	1 (12.5%)	
	CsA + MMF + PDN	15 (10.3%)	14 (11.7%)	1 (5.8%)	0	
Post-transplant infections	HCV	8 (5.5%)	8 (6.7%)	0	0	0.59
	CMV	41 (28.3%)	34 (28.3%)	4 (23.5%)	3 (37.5%)	0.62
	Bacterial	48 (33.1%)	35 (29.2%)	8 (47.1%)	5 (62.5%)	0.07

*Quantitative variables are displayed with median and standard deviation. Count variables are displayed raw and intragroup relative frequency. HCV, Hepatitis C Virus; HBV, Hepatitis B Virus; PBC, Primary Biliary Cholangitis; PSC, Primary Sclerosing Cholangitis; NASH, Non-Alcoholic Steatohepatitis; DBD, Donor after Brain Death; DCD, Donor after Cardiac Death; TAC, Tacrolimus; PDN, Prednisone; MMF, Mycophenolate Mofetil; CsA, Cyclosporine A; CMV, Cytomegalovirus*.

### Rejection Episodes

Eighteen episodes of TCMAR were diagnosed in 17 patients (one patient had two events). Regarding the severity, 9 were moderate, and 9 were mild. With respect to the timing, three episodes of TCMAR were diagnosed during the first week post-transplantation, six during the second week, four at the end of month 1, one at month 3, two at month 6, and two at month 12. All of these episodes were recovered with therapy, and no graft was lost due to rejection. Eight patients were diagnosed with SCR at the time of the per-protocol biopsy. Only one of these eight patients developed clinical TCMAR, after the follow-up period expired. As reflected in Table 1, no significant differences were found in the main characteristics of the cohort when comparing those patients who did not develop rejection, patients who presented TCMAR and those with SCR.

### Pharmacokinetics

Trough concentrations for TAC and CsA, and MPA and prednisone doses are summarized in Table 2. No statistically significant differences between rejectors (TCMAR or patients with SCR) and non-rejectors were observed in trough concentrations for TAC or CsA neither in MPA or prednisone doses during the period evaluated.

**Table 2:** Pharmacokinetics parameters.

	**No AR (120)**	**TCMAR (*n* = 17)**	**SCR (*n* = 8)**	**No AR vs. TCMAR**	**No AR vs. SCR**	**TCMAR vs. SCR**
	**1st Week**	**1st Week**	**1st Week**	***P* value**	***P* value**	***P* value**
TAC dose (mg/day)	4.0 ± 2.3	4.5 ± 2.1	5.0 ± 2.0	0.739	0.793	0.889
Cmin TAC (ng/mL)	5.5 ± 3.5	5.0 ± 3.4	6.0 ± 2.4	0.779	0.894	0.961
CsA dose (mg/day)	300.0 ± 142.1	650.0 ± 212.1	500.0 ± 125.3	0.059	0.333	0.667
Cmin CsA (ng/mL)	177.0 ± 83.9	105.6 ± 67.1	125.3 ± 56.4	0.134	0.083	0.500
Prednisone (mg/day)	20 ± 4.1	20 ± 4.3	20 ± 6.5	0.753	0.398	0.448
MPA dose (mg/day)	2000 ± 468.1	2000 ± 333.3	2000 ± 250.5	0.424	0.616	0.909
	**No AR (123)**	**TCMAR (*****n*** **=** **14)**	**SCR (*****n*** **=** **8)**	**No AR vs. TCMAR**	**No AR vs. SCR**	**TCMAR vs. SCR**
	**Day 15th**	**Day 15th**	**Day 15th**	***P*****-value**	***P*****-value**	***P*****-value**
TAC dose (mg/day)	6.0 ± 2.9	7.0 ± 1.9	5.5 ± 1.8	0.661	0.703	0.571
Cmin TAC (ng/mL)	5.8 ± 2.3	4.9 ± 1.5	5.2 ± 1.9	0.339	0.811	0.827
CsA dose (mg/day)	400.0 ± 110.7	500.0 ± 141.4	395.0 ± 105.2	0.333	0.500	0.667
Cmin CsA (ng/mL)	144.0 ± 91.2	186.3 ± 10.9	150.2 ± 11.5	0.436	0.200	0.667
Prednisone (mg/day)	20 ± 1.1	20 ± 1.0	20 ± 0.5	0.843	0.919	1.000
MPA dose (mg/day)	2000 ± 522.4	2000 ± 408.2	2000 ± 350.5	0.733	0.589	0.857
	**No AR (129)**	**TCMAR (*****n*** **=** **8)**	**SCR (*****n*** **=** **8)**	**No AR vs. TCMAR**	**No AR vs. SCR**	**TCMAR vs. SCR**
	**1st Month**	**1st Month**	**1st Month**	***P*****-value**	***P*****-value**	***P*****-value**
TAC dose (mg/day)	8.0 ± 2.8	4.0 ± 2.9	4.5 ± 0.7	**0.030**	**0.045**	0.800
Cmin TAC (ng/mL)	7.1 ± 2.4	5.5 ± 2.3	6.6 ± 1.9	0.608	0.833	0.573
CsA dose (mg/day)	300.0 ± 122.4	400.0 ± 120.2	300. ± 1.77.2	0.600	0.400	0.317
Cmin CsA (ng/mL)	194.2 ± 109.2	238.6 ± 65.5	225.5 ± 56.3	0.870	0.273	0.667
Prednisone (mg/day)	20 ± 2.3	20 ± 2.2	20 ± 2.5	0.957	0.913	0.905
MPA dose (mg/day)	2000 ± 543.3	2000 ± 520.3	2000 ± 495.6	0.246	0.246	0.958
	**No AR (133)**	**TCMAR (*****n*** **=** **4)**	**SCR (*****n*** **=** **8)**	**No AR vs. TCMAR**	**No AR vs. SCR**	**TCMAR vs. SCR**
	**2nd Month**	**2nd Month**	**2nd Month**	***P*****-value**	***P*****-value**	***P*****-value**
TAC dose (mg/day)	6.0 ± 2.9	4.0 ± 1.9	4.5 ± 2.1	0.130	0.104	0.48
Cmin TAC (ng/mL)	7.3 ± 2.8	5.2 ± 2.0	6.6 ± 2.5	0.133	0.213	0.221
CsA dose (mg/day)	200.0 ± 98.5	400 ± 135.2	150.0 ± 95.6	0.308	0.462	0.317
Cmin CsA (ng/mL)	184.0 ± 53.3	310.9 ± 98.5	165.2 ± 44.6	0.143	0.714	0.321
Prednisone (mg/day)	15 ± 4.4	12.5 ± 3.5	15 ± 4.1	0.459	0.946	0.533
MPA dose (mg/day)	1500 ± 535.9	2000 ± 126.3	2000 ± 545.6	0.216	0.216	0.901
	**No AR (133)**	**TCMAR (*****n*** **=** **4)**	**SCR (*****n*** **=** **8)**	**No AR vs. TCMAR**	**No AR vs. SCR**	**TCMAR vs. SCR**
	**3rd Month**	**3rd Month**	**3rd Month**	***P*****-value**	***P*****-value**	***P*****-value**
TAC dose (mg/day)	7.0 ± 3.1	5.9 ± 2.3	5.0 ± 2.2	0.157	0.549	0.317
Cmin TAC (ng/mL)	7.6 ± 2.9	7.5 ± 3.9	5.8 ± 1.5	0.978	**0.046**	0.329
CsA dose (mg/day)	247.0 ± 98.5	250 ± 78.5	150.0 ± 89.5	0.364	0.364	0.966
Cmin CsA (ng/mL)	184.5 ± 56.6	239.1 ± 113.8	177.5 ± 54.2	**0.032**	0.909	0.667
Prednisone (mg/day)	10 ± 4.5	7.5 ± 3.5	10 ± 5.0	0.288	0.191	0.200
MPA dose (mg/day)	1000 ± 494.5	2000 ± 175.5	2000 ± 495.5	0.062	0.055	0.933
	**No AR (134)**	**TCMAR (*****n*** **=** **3)**	**SCR (*****n*** **=** **8)**	**No AR vs. TCMAR**	**No AR vs. SCR**	**TCMAR vs. SCR**
	**6th Month**	**6th Month**	**6th Month**	***P*****-value**	***P*****-value**	***P*****-value**
TAC dose (mg/day)	5.0 ± 2.7	4.5 ± 2.3	5.0 ± 2.1	0.444	0.810	0.756
Cmin TAC (ng/mL)	7.5 ± 2.2	8.5 ± 5.7	6.5 ± 2.0	0.752	0.326	0.982
CsA dose (mg/day)	250.0 ± 58.5	300 ± 0	150.0 ± 75.9	0.126	0.200	0.317
Cmin CsA (ng/mL)	149.0 ± 53.1	185.5 ± 0	173.4 ± 61.5	0.800	0.827	0.735
Prednisone (mg/day)	5 ± 3.8	3.5 ± 1.5	5 ± 4.5	0.165	0.209	0.500
MPA dose (mg/day)	1000 ± 378.2	Ø	1500 ± 288.6	Ø	0.125	Ø
	**No AR (134)**	**TCMAR (*****n*** **=** **3)**	**SCR (*****n*** **=** **8)**	**No AR vs. TCMAR**	**No AR vs. SCR**	**TCMAR vs. SCR**
	**9th Month**	**9th Month**	**9th Month**	***P*****-value**	***P*****-value**	***P*****-value**
TAC dose (mg/day)	5.0 ± 2.1	3.0 ± 0	4.0 ± 2.6	0.069	0.089	0.835
Cmin TAC (ng/mL)	7.5 ± 1.9	7.7 ± 0	8.3 ± 0.9	0.966	0.422	0.655
CsA dose (mg/day)	200 ± 46.9	200 ± 0	155.0 ± 65.5	0.985	0.250	0.715
Cmin CsA (ng/mL)	137.0 ± 40.63	170.2 ± 0	163.4 ± 69.6	0.525	0.126	0.791
Prednisone (mg/day)	5 ± 4.3	5 ± 2.5	5 ± 2.5	0.647	0.724	0.800
MPA dose (mg/day)	720 ± 422.9	Ø	1000 ± 288.7	Ø	0.178	Ø
	**No AR (135)**	**TCMAR (*****n*** **=** **2)**	**SCR (*****n*** **=** **8)**	**No AR vs. TCMAR**	**No AR vs. SCR**	**TCMAR vs. SCR**
	**12th Month**	**12th Month**	**12th Month**	***P*****-value**	***P*****-value**	***P*****-value**
TAC dose (mg/day)	5.0 ± 2.1	3.0 ± 0	3.8 ± 0.4	0.375	0.273	0.667
Cmin TAC (ng/mL)	6.7 ± 2.4	12.3 ± 0	6.0 ± 2.2	0.125	0.742	0.221
CsA dose (mg/day)	200.0 ± 77.3	200 ± 0	150.0 ± 48.6	0.958	0.526	0.712
Cmin CsA (ng/mL)	144 ± 30.3	104.2 ± 0	179.5 ± 32.5	0.750	0.325	0.625
Prednisone (mg/day)	0	0	0	0.667	0.886	0.500
MPA dose (mg/day)	860 ± 190.4	Ø	1000 ± 250.3	Ø	0.727	Ø

*Bold value of P ≤ 0.05 was considered significant*.

### miRNA Expression and TCMAR

**Figure 1:**
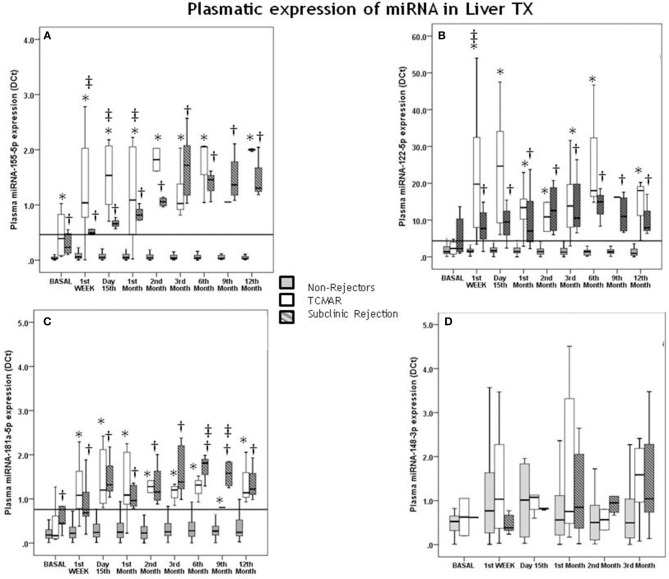
Correlation of pre- and post-transplantation plasmatic miRNA expression with acute rejection (TCMAR) and subclinical rejection (SCR). Differences between TCMAR patients (white boxes), non-rejectors (gray boxes) and subclinical rejectors (gray hatched boxes) with respect to miR-155-5p **(A)**, miR-122-5p **(B)**, miR-181a-5p **(C)**, and miR-148-3p expression **(D)**, over 1 year post-transplantation. Seventeen of the 145 patients experienced TCMAR episodes (3 episodes occurred during the 1st week post-transplantation, 6 at day 15th, 4 at the end of the 1st month, 1 during the 3rd month, 1 during the 6th month, and 2 during the 12th month post-transplantation). Eight patients were diagnosed with SCR and were considered an independent group. TCMAR box-plots include data from the patients who exhibited rejection at this time plus the pre-TCMAR data of the patients who had not yet exhibited rejected at this time but who would do so in a later profile. Therefore, the number of samples that contributed to the data for both groups in each profile is as follows: non-rejectors: pre-transplantation *n* = 120; 1st week *n* = 120; 15th day *n* = 123; 1st month *n* = 129; 2nd month *n* = 133; 3rd month *n* = 133; 6th month *n* = 134; 9th month *n* = 135 and 12th month *n* = 135 vs. TCMAR rejectors pre-transplantation *n* = 17; 1st week *n* = 17; 15th day *n* = 14; 1st month *n* = 8; 2nd month *n* = 4; 3rd month *n* = 4; 6th month *n* = 3; 9th month *n* = 2 and 12th month *n* = 2. *Indicates significant differences between TCMAR and non-rejectors; †Indicates significant differences between SCR and non-rejectors; and ‡Indicates significant differences between TCMAR and patients with SCR. The solid horizontal line indicates the post-transplantation cut-off value (0.453) for miR-155-5p expression **(A)**, the post-transplantation cut-off value (4.356) for miR-122-5p expression **(B)**, the post-transplantation cut-off value (0.760) for miR-181a-5p expression **(C)**.

Pre-transplantation, no significant differences were observed in the plasmatic expression of miR-122-5p, miR-181a-5p, and miR-148-3p between patients who suffered a TCMAR rejection episode after transplantation from those patients who were free of rejection (Figures 1B–D). Only the pre-transplantation differences between both groups achieved significance for the expression of miR-155-5p (*P* < 0.001) (Figure 1A).

Post-transplantation, a significant increase in the expression of miR-155-5p, miR-122-5p, and miR-181a-5p was observed in rejector patients compared with that in non-rejector patients (Figures 1A–C); however, no significant differences were observed in the plasmatic expression of miR-148-3p (Figure 1D).

**Figure 2:**
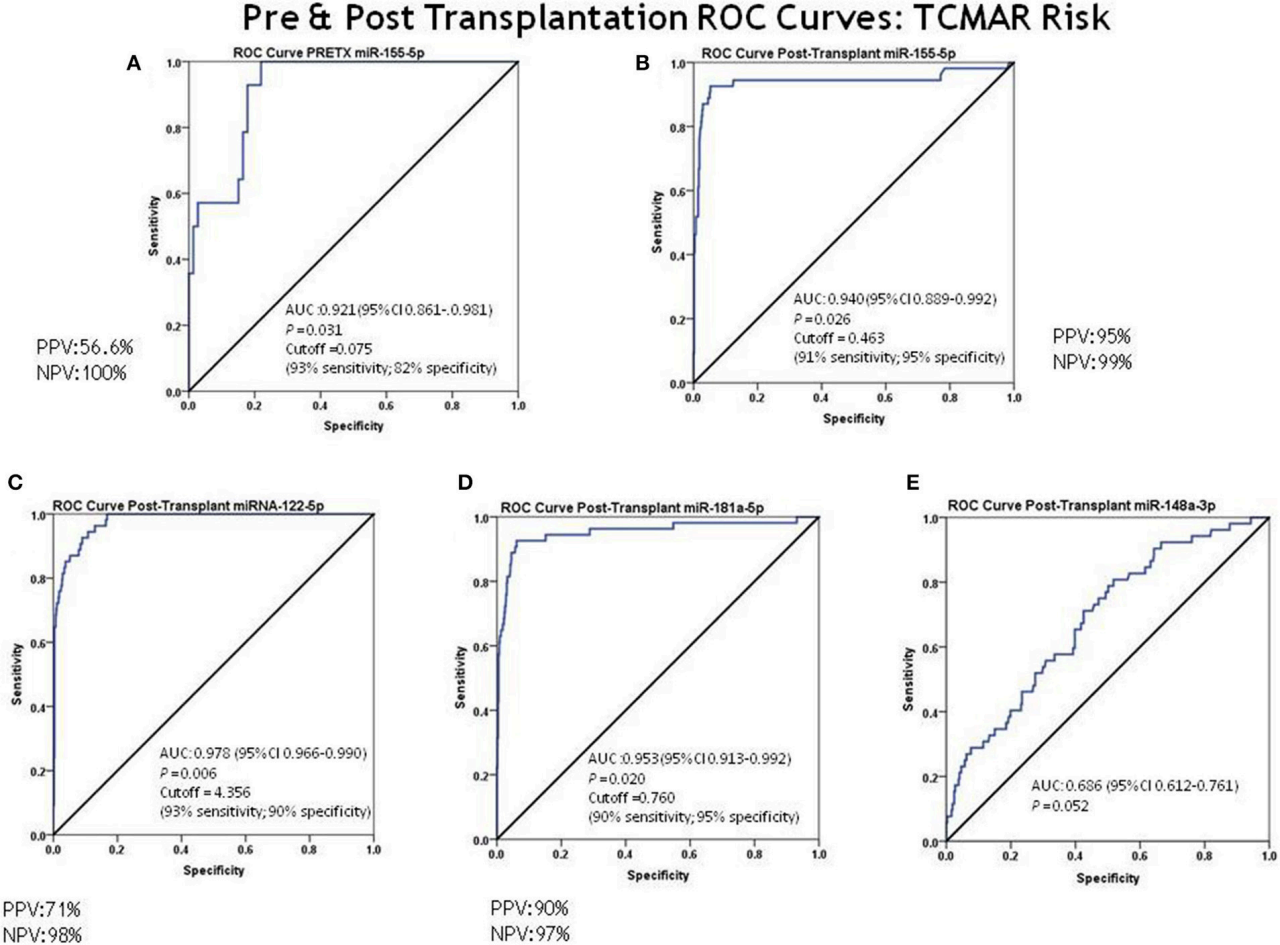
ROC curve analysis for discrimination between rejectors (TCMAR) and non-rejectors. ROC curve analysis for discrimination between rejectors (TCMAR) and non-rejectors for each miRNA was evaluated. **(A)** Pre-transplantation miR-155-5p capacity to discriminate between rejectors and non-rejectors (AUC = 0.921; 95% CI = 0.861–0.981; cut-off value = 0.075 [93% sensitivity, 82% specificity, PPV = 56.6% and NPV = 100%)]; **(B–E)** Post-transplantation miRNA capacity to discriminate between rejectors and non-rejectors: **(B)** miRNA-155-5p AUC = 0.940 (95% CI = 0.889–0.992) and cut-off value = 0.463 (91% sensitivity, 95% specificity, PPV = 95% and NPV = 99.8%); **(C)** miR-122-5p AUC = 0.978 (95% CI = 0.966–0.990) and cut-off value = 4.356 (93% sensitivity, 90% specificity, PPV = 71% and NPV = 98%); **(D)** miRNA-181a-5p AUC = 0.953 (95% CI = 0.913–0.992) and cut-off value = 0.760 (90% sensitivity, 95% specificity, PPV = 90% and NPV = 97%); **(E)** miRNA-148-3p AUC = 0.686 (95% CI = 0.612–0.761).

Cut-off values for prognostic TCMAR were determined based on the AUC of the ROC curve analysis for each miRNA, with significantly higher levels in patients who showed rejection (TCMAR). ROC curve analysis showed that pre-transplantation, miR-155-5p had an outstanding capacity to discriminate between rejectors and non-rejectors (AUC = 0.921; 95% CI = 0.861–0.981) (Figure 2A). The optimal cut-off value for prognostic TCMAR based on the AUC of the ROC curve for miR-155-5p was 0.075, with 93% sensitivity, 82% specificity, 56.6% PPV, and 100% NPV (Supplementary Table 1).

Post-transplantation, ROC curve analysis showed that miR-155-5p, miRNA-122-5p, and miRNA-181a-5p also showed an outstanding capacity to discriminate between rejectors and non-rejectors: miRNA-155-5p, AUC = 0.940 (95% CI = 0.889–0.992), an optimal cut-off value to prognostic TCMAR of 0.463 with a 91% sensitivity, 95% specificity, 95% PPV, and 99% NPV (Figure 2B); miR-122-5p, AUC = 0.978 (95% CI = 0.966–0.990), an optimal cut-off value to prognostic TCMAR of 4.356 with a 93% sensitivity, 90% specificity, 71% PPV, and 98% NPV (Figure 2C); and miR-181a-5p, AUC = 0.953 (95% CI = 0.913–0.992), an optimal cut-off value to prognostic TCMAR of 0.760 with a 90% sensitivity, 95% specificity, 90% PPV, and 97% NPV (Figure 2D). In contrast, miR-148-3p showed a low capacity to discriminate between rejectors and non-rejectors (AUC = 0.686; 95% CI = 0.612–0.761) (Figure 2E) (Supplementary Table 1).

**Figure 3:**
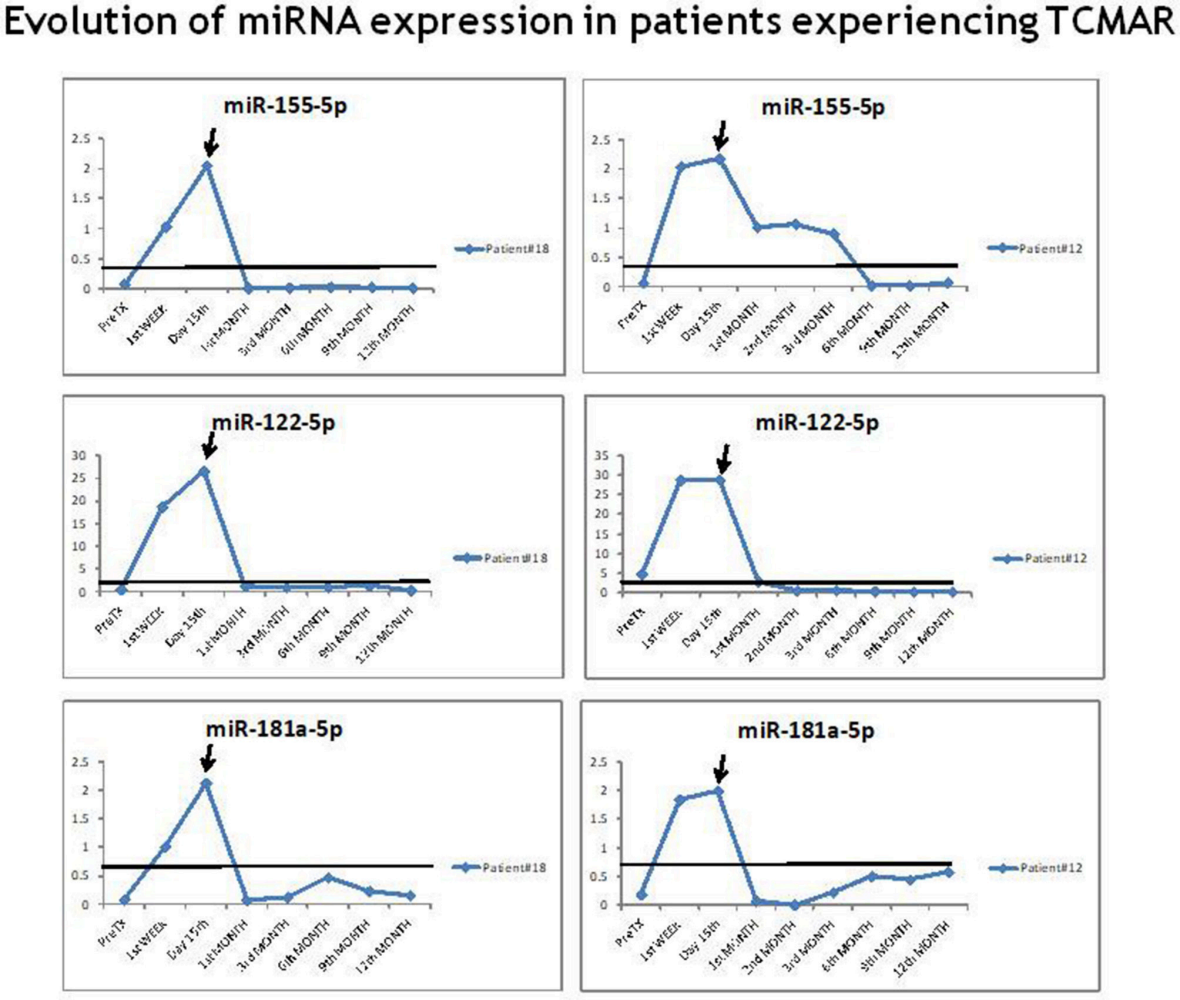
Evolution of miR-155-5p, miR-122-5p, and miR-181a-5p expression in patients experiencing TCMAR. An example of the evolution of the miR-155-5p, miR-122-5p, and miR-181a-5p expression in two rejector patient prior to, during and after the TCMAR episodes. The arrow indicates the time of the TCMAR episode.

The results of an analysis of the individual evolution in the expression of miR-155-5p, miR-122-5p, and miR-181a-5p in each rejector patient prior to, during and after the TCMAR episodes showed that the expression of each miRNA progressively increased preceding the TCMAR episode and reached maximum levels at the time of the episode. Once the immunosuppressive treatment was modified to resolve the TCMAR episode, the miRNA levels decreased (Figure 3). Comparing the miRNA expression evolution with the evolution of serum aminotransferase levels, and taking into account that aminotransferase levels were not normalized until 1–2 weeks post-surgery, in patients who exhibited rejection after 15 days of transplantation (8 of 17 patients), the increase in the plasmatic miR-155-5p, miR-122-5p, and miR-181a-5p expression occurred before aminotransferase levels were modified during TCMAR. Specifically, in patients who showed rejection at 3, 6, or 12 months post-transplantation, these miRNAs levels were above the cut-off value for the risk of rejection in the first weeks post-transplantation, and their increase was earlier and more rapid than the serum levels of aspartate aminotransferase (AST), alanine aminotransferase (ALT), and gamma-glutamyl-transpeptidase (GGT), which usually took place 2–3 weeks before rejection occurred. Furthermore, no significant differences were observed in the evolution of the serum aminotransferase levels between non-rejectors, TCMAR and SCR patients (Supplementary Table 2).

### miRNA Expression and SCR

Pre-transplantation differences between non-rejectors and patients who were diagnosed with SCR after transplantation achieved significance for the expression of miR-155-5p (*P* < 0.001) (Figure 1A) and miRNA-181a-5p (*P* = 0.012) (Figure 1C); however, no significant differences were observed in the plasmatic expression of miR-122-5p and miR-148-3p between SCR patients and those free of rejection (Figures 1B,D).

Post-transplantation, a significant increase in the expression of miR-155-5p, miR-122-5p, and miR-181a-5p was observed in patients with SCR compared with that in non-rejector patients (Figures 1A–C); however, no significant differences were observed in the plasmatic expression of miR-148-3p (Figure 1D).

**Figure 4:**
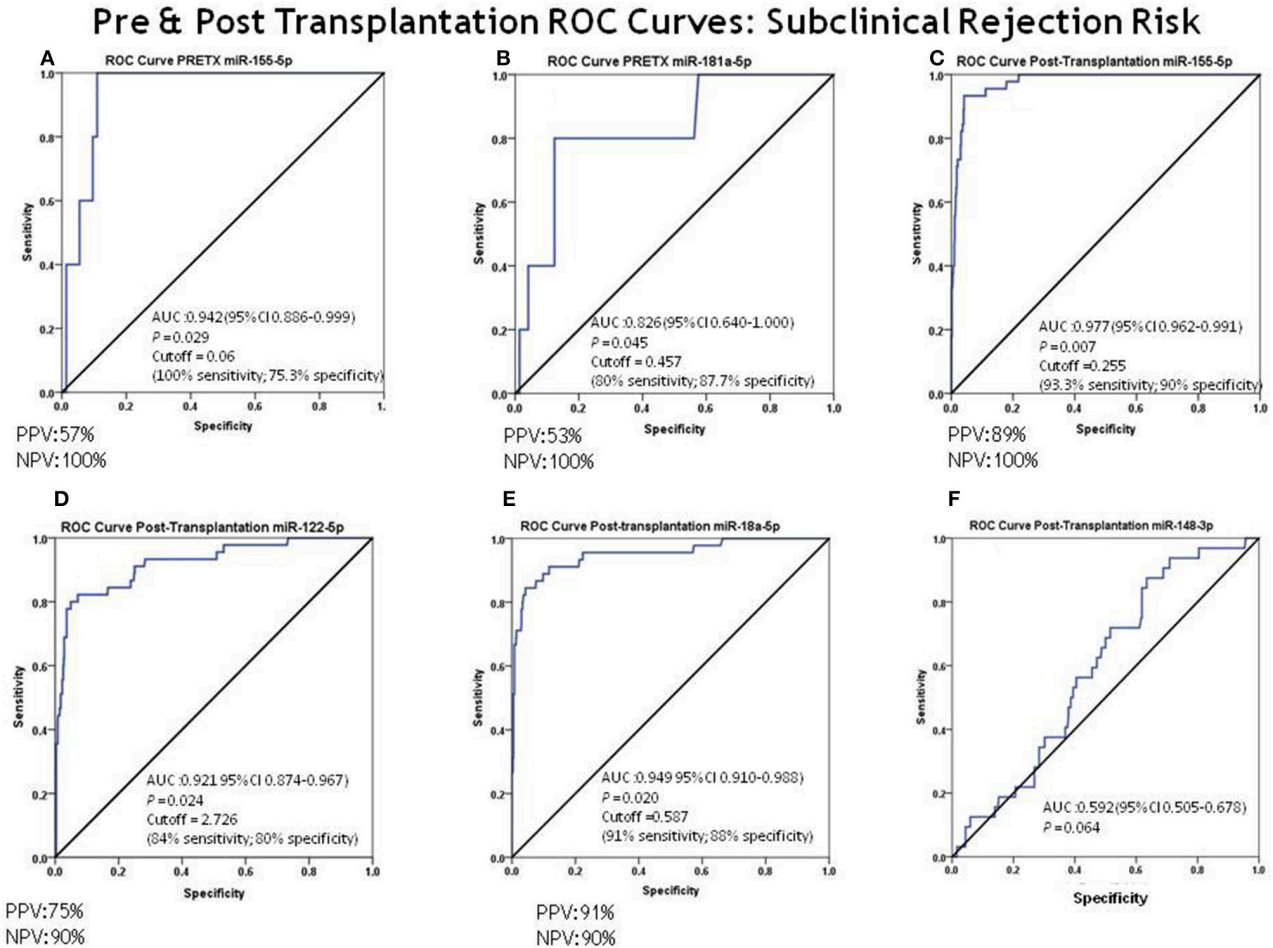
ROC curve analysis for discrimination between subclinical rejectors (SCRs) and non-rejectors. The ROC curve analysis for discrimination between patients with SCR and non-rejectors for each miRNA was evaluated. **(A)** Pre-transplantation miR-155-5p capacity to discriminate between SCR and non-rejectors: AUC = 0.942 (95% CI = 0.886–0.999) and cut-off value = 0.06 (100% sensitivity, 75.3% specificity, PPV = 57% and NPV = 100%); **(B)** pre-transplantation miR-181a-5p capacity to discriminate between SCR and non-rejectors: AUC = 0.826 (95% CI = 0.640–1.000) and cut-off value = 0.457 (80% sensitivity, 87.7% specificity, PPV = 53% and NPV = 100%); **(C–F)** post-transplantation miRNA capacity to discriminate between SCR and non-rejectors: **(B)** miR-155-5p AUC = 0.977 (95% CI = 0.962–0.991) and cut-off value = 0.255 (93.3% sensitivity, 90% specificity, PPV = 89% and NPV = 100%); **(C)** miR-122-5p: AUC = 0.921 (95% CI = 0.874–0.967) and cut-off value = 2.726 (84% sensitivity, 80% specificity, PPV = 75% and NPV = 90%); **(D)** miR-181a-5p: AUC = 0.949 (95% CI = 0.910–0.988) and cut-off value = 0.587 (91% sensitivity, 80% specificity, PPV = 91% and NPV = 90%); **(E)** miR-148-3p: AUC = 0.592 (95% CI = 0.505–0.678).

Cut-off values to prognostic SCR were also determined based on the AUC of the ROC curve analysis for each miRNA, with significantly higher levels in patients with SCR (Figure 4). Pretransplantation, miR-155-5p had an outstanding capacity to discriminate between SCR patients and non-rejectors (AUC = 0.942; 95% CI = 0.886–0.999) (Figure 4A). The optimal cut-off value to prognostic SCR based on the AUC of the ROC curve for miR-155-5p was 0.060, with 100% sensitivity, 75.3% specificity, 57% PPV, and 100% NPV. For miR-181a-5p, the results showed an excellent capacity to discriminate between SCR patients and non-rejectors (AUC = 0.826; 95% CI = 0.640–1.000) (Figure 4B). The optimal cut-off value to prognostic SCR based on the AUC of the ROC curve for miR-181a-5p was 0.457, with 80% sensitivity, 87.7% specificity, 53% PPV, and 100% NPV (Supplementary Table 1).

Post-transplantation, ROC curve analysis showed that miR-155-5p, miR-122-5p, and miR-181a-5p also had an outstanding capacity to discriminate between SCR patients and non-rejectors: miR-155-5p, AUC = 0.977 (95% CI = 0.962–0.991), an optimal cut-off value to prognostic SCR of 0.255 with a 93.3% sensitivity, 90% specificity, 89% PPV, and 100% NPV (Figure 4C); miR-122-5p, AUC = 0.921 (95% CI = 0.874–0.967), an optimal cut-off value to prognostic SCR of 2.726 with an 84% sensitivity, 80% specificity, 75% PPV, and 90% NPV (Figure 4D); and miR-181a-5p, AUC = 0.949 (95% CI = 0.910–0.988), an optimal cut-off value to prognostic SCR of 0.587 with a 91% sensitivity, 88% specificity, 91% PPV, and 90% NPV (Figure 4E). miR-148-3p also showed a low capacity to discriminate between both groups of patients (AUC = 0.592; 95% CI = 0.505–0.678) (Figure 4F) (Supplementary Table 1).

**Figure 5:**
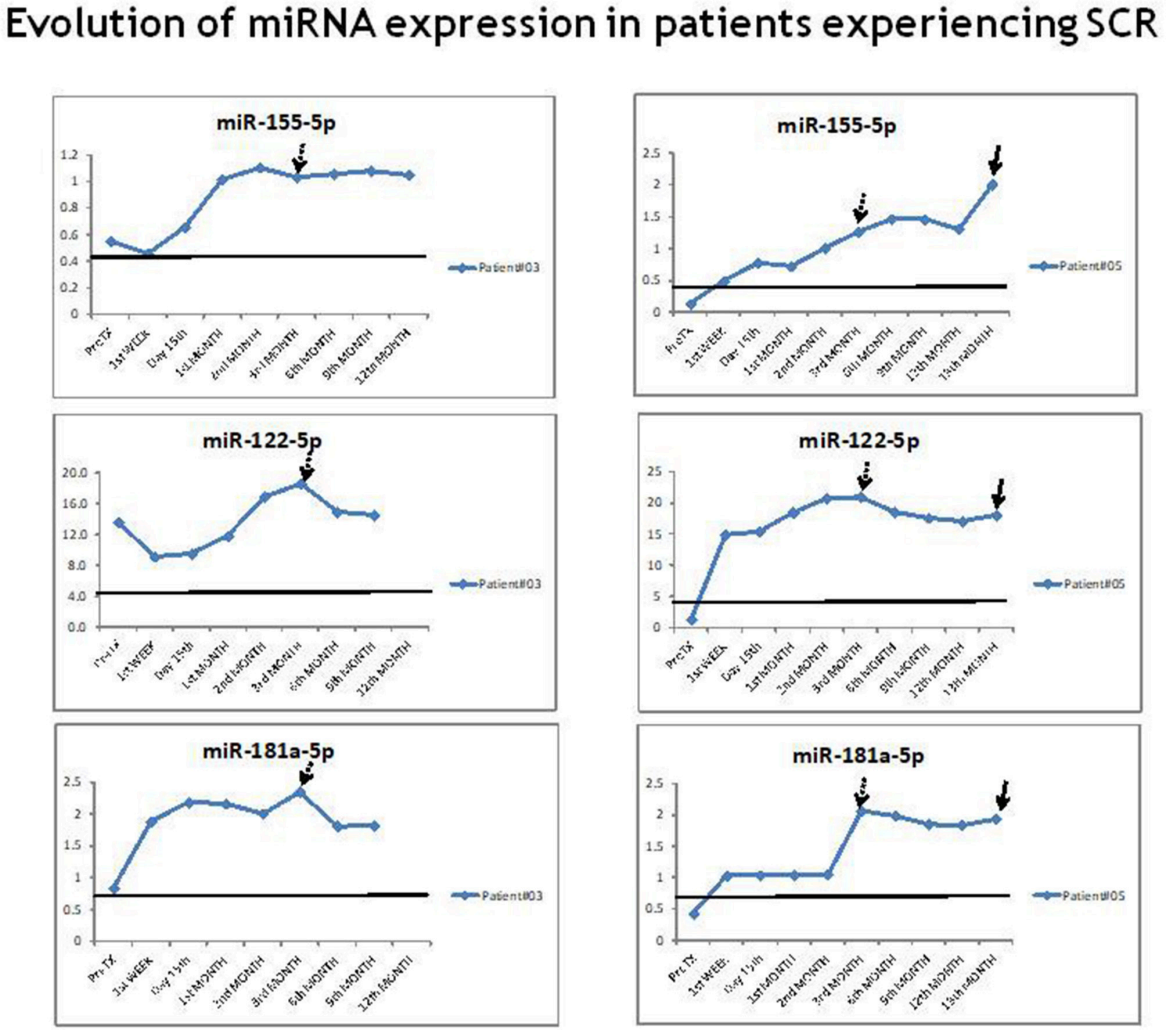
Evolution of miR-155-5p, miR-122-5p, and miR-181a-5p expression in patients experiencing subclinical rejection. An example of the evolution of the miR-155-5p, miR-122-5p, and miR-181a-5p expression in two patients with SCR. The arrow at 3 months post-transplantation indicates the time of the protocol biopsy. In patient #5, the second arrow indicates the time of the TCMAR episode.

In patients diagnosed with SCR during the protocol biopsy, the expression of each miRNA (miR-155-5p, miR-122-5p, and miR-181a-5p) also progressively increased, from pre-transplantation to the 3rd month post-transplantation, and it remained above the cut-off value for the risk of TCMAR during all periods of the study (1 year post-transplantation) (Figure 5). Instead, in all of these patients, after the 1st week post-transplantation, the serum transaminase levels remained within normal range. One of these patients (Patient #5) finally showed rejection at the 13th month post-transplantation, for which we obtained a sample to evaluate miRNA expression (Figure 5). While their miRNA levels were already elevated after the immediate post-transplant period, their transaminase values did not increase until 3 weeks before the TCMAR occurred.

### miRNA Expression in Patients With SCR and TCMAR

Between rejectors (TCMAR) and patients with SCR, differences in the expression of miR-155-5p were significantly increased in TCMAR until the 2nd month post-transplantation, after which no significant differences were observed in either group (Figure 1A). For miR-122-5p expression, except in the 1st week post-transplantation, no differences were observed in either group (Figure 1B). Additionally, for miR-181a-5p after the 3rd month post-transplantation, SCR patients showed an increase in expression compared with the TCMAR group, and these differences disappeared 1 year post-transplantation (Figure 1C).

### Development and Validation of a Prognostic Algorithm for the Risk of TCMAR and SCR Based on miR-155-5p and miR-181a-5p Expression

A binary logistic regression model was performed to assess the prognostic capacity of the risk of TCMAR or SCR events based on the expression of miR-155-5p and miR-181a-5p individually or in combination as explanatory variables.

The results showed that TCMAR risk was better predicted by the individual expression of miR-181a-5p than by miR-155-5p alone or both miRNAs in combination. Concerning SCR risk prediction, the model that considers a combination of both miRNAs showed a high capacity to prognostic this clinical event. Bootstrap analysis, after 1,000 resamplings, and a vpc, after 1,000 simulations, demonstrated the robustness of the TCMAR and SCR models. The estimated parameters, logit function and bootstrap results of both models are shown in Table 3.

**Table 3:** Logistic regression model parameters.

**Parameter**	**Estimation (RSE%)**	**Bootstrap (P2.5–P97.5)**
**(A) TCMAR LOGISTIC REGRESSION MODEL**		
ß_0_	−6.35 (9%)	−6.65 (−8.17 to −4.52)
ß_1_	3.87 (14%)	4.16 (1.92 to 5.82)
MOFV	86.324	
	LOGIT = −6.35 + 3.87 * miR-181a-5p		
**(B) SRC LOGISTIC REGRESSION MODEL**		
ß_0_	−5.18 (9%)	−5.31 (−6.29 to −4.07)
ß_1_	2.27 (33%)	2.28 (0.27 to 4.27)
ß_2_	1.74 (36%)	1.80 (0.07 to −3.41)
MOFV	153.787	
	LOGIT = −5.18 + 2.27*miR-181a-5p + 1.74*miR-155-5p		

### Correlation Between miRNA Expression and HCV Infection

**Figure 6:**
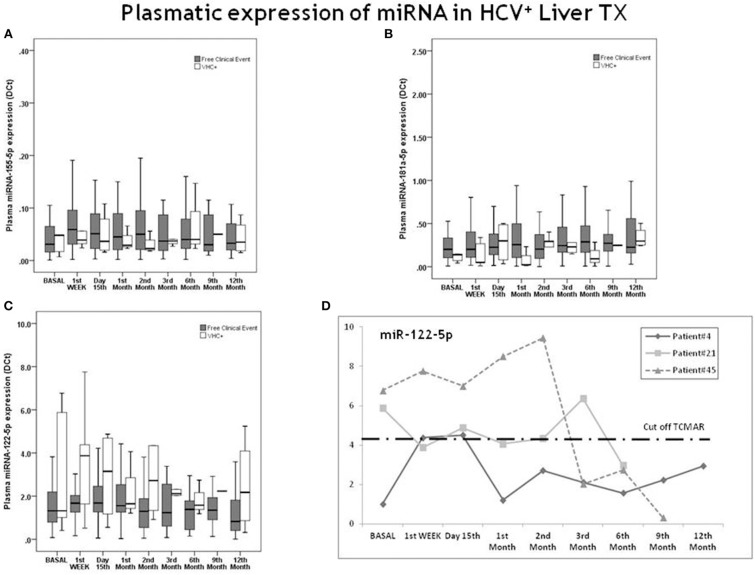
Evolution of miR-155-5p, miR-122-5p, and miR-181a-5p expression in patients experiencing the recurrence of HCV^+^. Differences between patients free of any clinical event (gray boxes) and HCV^+^ patients (white boxes) with respect to **(A)** miR-155-5p; **(B)** miR-181a-5p and **(C)** miR-122-5p expression during 1 year post-transplantation. Five patients developed HCV recurrence after transplantation (*n* = 3 HCV RNA^+^ patients until the 3rd month post-transplantation, with exitus occurring in one of them; one patient, until the 2nd month; and one patient, until the 6th month). Graph **(D)** shows the individual evolution in miR-122-5p expression in 3 patients with HCV^+^. The dashed horizontal line indicates the post-transplantation cut-off value for the risk of TCMAR (4.356) with respect to miR-122-5p expression.

None of the transplanted patients with positive HCV RNA developed rejection. Seven of them were treated with direct antivirals during first year after LT (obtaining sustained viral response in 6 of them), while one persisted with positive RNA at the end of follow up. We analyzed their miRNA profile expression, and the results showed no significant differences with the remaining non-rejector patients (Figure 6). Individuals with HCV recurrence showed plasma levels of miR-155-5p and miR-181a-5p below the cut-off values established for the risk of TCMAR. In the case of miR-122-5p, the results showed a trend to be higher in the HCV^+^ group with respect to non-rejectors, but this difference was not statistically significant (Figure 6C). However, when we analyzed the miRNA expression individually, in 3 of 8 HCV^+^ patients the results showed miR-122-5p plasma levels above the cut-off value established for the risk of TCMAR during the period with detectable RNA (Figure 6D). Therefore, for this specific miRNA, the presence of an active HCV infection could be a confounding factor for predicting rejection.

## Discussion

To our knowledge, this study is the first to monitor the plasma expression of a panel of miRNAs sequentially in time, pre-transplantation and over one year post-transplantation (1st week, day 15th and after the 1st, 2nd, 3rd, 6th, 9th, and 12th month) in *de novo* liver recipients. This design provides a better evaluation of the changes in the expression of the evaluated miRNAs and its prognostic capacity for the risk of TCMAR. Furthermore, this study is the first to evaluate a panel of plasmatic miRNAs as a potential biomarker for the assessment of the risk of SCR in LT.

This study demonstrates that sequential monitoring of plasmatic miRNA expression levels could reveal useful prognostic and diagnostic biomarkers for TCMAR and SCR during the post-transplantation period. Specifically, we found that the plasmatic expression of miR-181a-5p, miR-155-5p, and miR-122-5p were statistically significant higher in TCMAR and in SCR patients compared with non-rejectors and could diagnose and prognostic TCMAR and SCR with high accuracy. However, no differences in either group of patients were observed for miR-148-3p expression.

Although the results showed that pre-transplantation, miR-155-5p expression was significantly higher in rejectors (TCMAR) and in the SCR group than in non-rejectors, and miR-181a-5p expression was higher in SCR patients than in non-rejectors, and the ROC curve analysis showed an excellent AUC value, the discrete PPV values (~50%) did not allow for accurately discriminating patients at risk of suffering clinical events from false positive cases. However, the method appears capable of accurately identifying those patients without risk of suffering TCMAR and SCR, because its NPV was 100%, and as a consequence, those patients with an expression below the established cut-off could be candidates to receive IS therapy that is more moderate, minimizing the adverse effects associated with treatment.

Post-transplantation, our results showed a significant increase in the expression of miR-181a-5p, miR-155-5p, and miR-122-5p in patients who experienced rejection (TCMAR). The AUC (>0.940), PPV (>70%), and NPV (>95%) values for these miRNAs were excellent, indicating a very good discriminatory ability to identify patients at high risk of developing TCMAR. Our results are in line with previous reports on the potential of miRNAs as diagnostic biomarkers of AR (10, 16, 23). In the case of miR-155-5p, our group demonstrated that this miRNA could be a useful prognostic biomarker for the risk of TCMAR in both liver and renal transplant recipients (8). In the case of patients with SCR, the results also showed a significant increase in the expression of miR-155-5p and miR-122-5p, but lower than that in TCMAR patients, compared with the expression in patients free of any clinical event. miR-181a-5p expression in the SCR group reached even higher levels than that in the TCMAR group from the third month post-transplantation; nevertheless, these differences disappeared 1 year post-transplantation. Li et al. demonstrated that the TCR sensitivity and signaling strength can be modulated by this miRNA and thus may be critical for regulating the development of effector cell function (25). The AUC (>0.820), PPV (>70%), and NPV (>90%) values for these miRNAs were excellent, indicating a very good discriminatory ability to identify patients at high risk of developing SCR.

The fact that in each TCMAR patient, the individual expression of miR-181a-5p, miR-155-5p, and miR-122-5p gradually was upregulated prior to the TCMAR episode (and earlier than serum transaminases levels were modified during the AR) and reached maximum levels at the time of the episode, clearly shows not only a diagnostic capacity of the miRNAs but also their potential utility as non-invasive prognostic biomarkers. Many previous studies have reported a marked elevation of the transaminase levels in the first hours after liver reperfusion, with a peak on the 1st or 2nd day after transplantation and, unless an episode of acute rejection occurs, they are usually normalized within the first 15 days post-transplantation (34). Therefore, the elevation of these biochemical parameters during the first days post-transplantation is not specific for a rejection diagnosis because there may be other complications that also alter them. It is important to note that, in our study population, those patients who showed rejection exhibited an increase in miR-155-5p, miR-122-5p, and miR-181a-5p expression that was earlier and more rapid than the increase in serum transaminase levels from the first weeks post-transplantation. Furthermore, we observed that in patients with SCR, in contrast to the serum transaminase levels, which remained within the normal range in all the periods evaluated, the expression of miR-181a-5p, miR-155-5p, and miR-122-5p increased progressively from pre-transplantation to the 3rd month post-transplantation. However, in this case, the expression levels kept increasing above the cut-off value established in this study for the risk of TCMAR during the entire period of the study (1 year post-transplantation), indicating that an inflammatory process remained without resolving. In fact, one of these patients ended up experiencing rejection 1 month after finishing the study. Several renal transplantation studies have shown that the detection, usually by surveillance biopsies, of SCR has an impact on the long-term allograft outcome given that SCR can lead to chronic tubulointerstitial damage, chronic graft dysfunction, chronic rejection and graft loss (35–37). The pathways by which persistent inflammation initiates a fibrogenic response that destroys the renal parenchyma are well-known. In contrast, the role of SCR in the long-term allograft outcome in LT is not clear. Recently, Londoño et al. (5) demonstrated the importance of subclinical inflammatory lesions in long-term stable LT recipients. The clinical significance of these lesions and their response to immunosuppressive treatment is currently uncertain. For this reason, in this context, the clinical utility of a protocol liver biopsy remains controversial, given that it is an invasive method that may favor some severe adverse events, and the benefit that is obtained is not clear; consequently, many centers do not include this procedure in adult LT programs. If this clinical entity could be detected by non-invasive biomarkers, such as the expression of certain miRNAs, the performance of this type of biopsy could be avoided, which would allow for the more accurate selection of patients who are truly candidates for minimizing IS treatment without risk of suffering rejection and for providing the correct treatment of patients with SCR to decrease this alloreactivity.

The logistic regression results demonstrated that miR-181a-5p expression showed a greater prognostic power for the risk of TCMAR than the individual expression of miR-155-5p or both miRNAs in combination. On the other hand, to identify patients at risk of suffering SCR, the model that combined both miRNAs (miR-181a-5p and miR-155-5p) was more robust than the model evaluating the expression of each miRNA. It is evident that a single biomarker may not be sufficient to reflect all of the complexities associated with LT, and it is necessary to identify a panel of distinct non-invasive biomarkers that can detect the degree of alloreactivity of individuals and diagnose and prognostic graft dysfunction as well as injury. According to our results for evaluating the risk of TCMAR, the plasmatic expression of miR-181a-5p (better than that of miR-155-5p) could be a good candidate for inclusion in this panel of biomarkers. To identify patients at the risk of suffering SCR, it would be preferable to include both miRNAs in this panel.

It is important to note that this study evaluated this miRNA panel in an adult LT population that was practically free of HCV infection (only 8 patients who presented HCV RNA positivity at the time of transplantation developed HCV recurrence, and all of them were non-rejector patients, with the remaining patients free of this infection). Therefore, to evaluate the prognostic capacity of these miRNAs for the risk of TCMAR and SCR, clinical confounding factors associated with this clinical event can be discarded. However, it is important to clarify that in HCV^+^ patients, miR-122-5p expression showed a tendency to be higher than that in patients free of rejection, and during the period with active infection, some patients showed levels above the cut-off value established for the risk of TCMAR. Therefore, for this specific miRNA, the presence of an active HCV infection could be a confounding factor to prognostic rejection; in this situation, there is a requirement to test the HCV viral load to rule out patients without risk of rejection. Several previous studies have described significantly elevated serum levels of miR-122-5p in HCV^+^ patients (38, 39).

Considering the possible implementation of these biomarkers in the clinical setting routine, some aspects should be addressed. Clearly, the following advantages of plasma markers for the diagnosis and prognosis, in this case, of liver TCMAR or SCR are obvious: less invasive; involves minimal previous manipulation of the sample; and less costly compared with the gold standard method (biopsy). However, there is still more to learn and discover about miRNAs themselves as well as their interaction with their target genes, and, on the other hand, some methodological critical points in the detection of miRNAs should be resolved to avoid discrepancies between centers. There is a need for interlaboratory analytical cross-validation and comprehensive standardization of all analytical process (e.g., isolation, storage, measurement, and quantification protocols, the selection of housekeeping genes to correct the data to normalize the results, etc.). It is necessary to develop more robust methods and to improve their sensitivity, specificity and reproducibility as well as to define the cut-off values for these promising miRNAs in the context of prospective, randomized, multicenter clinical trials that allow transplant patient stratification associated with a specific clinical event, such as TCMAR or SCR.

Because our analysis is only a single-center study, confirmation by other centers is needed. Furthermore, our study has some limitations. The event size (TCMAR and SCR) is small; in fact, this is a limitation common to all studies of this type of biomarker that has been published to date. It is important to note that the patients in our hospital were treated with triple therapy, and furthermore, this study was the first in which that the study population was practically free of HCV infection. Both of these factors surely impact the allograft response and, consequently, the rate of rejection. However, a large number of samples was included in the present study, which makes us confident in our data and strengthens the importance of our findings because our results still reached significance despite the small sample size. Furthermore, we have internally validated the two algorithms obtained after the binary logistic regression by the bootstrap method, and the results demonstrated the robustness of both algorithms. The study was performed in a Caucasian population, and our findings should also be validated in a separate non-Caucasian population; furthermore, the absence of patients with antibody-mediated rejection (ABMR) in our cohort did not allow us to evaluate the prognostic capacity of these miRNAs for this clinical event. Nevertheless, despite these limitations, this prospective observational study suggests that the sequential pre- and post-transplantation monitoring of the plasma expression of miR-181a-5p, miR-155-5p, and miR-122-5p may well provide key prognostic biomarkers for the risk of TCMAR and (for first time evaluated) for the risk of SCR, and the variation in the level of these miRNAs occurs earlier and more rapidly than that of serum transaminase levels. Pre-transplantation, miR-155-5p expression could be a useful tool to stratify patients at low immunologic risk; furthermore, post-transplantation, the expression of miR-181a-5p and miR-155-5p could be candidates for inclusion in an early, non-invasive prognostic biomarker panel to prevent TCMAR or SCR.

## Author Contributions

OM performed the miRNA analyses, interpreted the data, performed the statistical analysis, and drafted the manuscript. PR and GC selected and followed the patients. LO and MS performed patient blood samples extractions. PF performed the miRNA analyses. VF performed the pharmacokinetic analysis. LQ performed the binary logistic regression and bootstrap analysis. MN selected and followed the patients and revised the manuscript for important intellectual content. MB conceived and designed the study, interpreted the data, drafted and revised the manuscript. All authors approved the final version of the manuscript for submission.

### Conflict of Interest Statement

The authors declare that the research was conducted in the absence of any commercial or financial relationships that could be construed as a potential conflict of interest.

## Abbreviations

BPAR: biopsy-proven acute rejection
CsA: cyclosporine A
CI: confidence interval
CMV: cytomegalovirus
CNi: calcineurin inhibitor
HCV: hepatitis C virus
IS: immunosuppression
LT: liver transplantation
miRNA: microRNA
MMF: mycophenolate mofetil
MPA: mycophenolic acid
NPV: negative predictive value
PBMC: peripheral blood mononuclear cell
PPV: positive predictive value
qPCR: quantitative real-time PCR
SCR: subclinical rejection
ROC: receiver operating characteristic
TAC: tacrolimus
TCMAR: T cell-mediated acute rejection
Th: T helper.
