# Supplementary material for: Monitoring of miR-181a-5p and miR-155-5p Plasmatic Expression as Prognostic Biomarkers for Acute and Subclinical Rejection in de novo Adult Liver Transplant Recipients
Source: Front Immunol. 2019 Apr 24;10:873. doi: 10.3389/fimmu.2019.00873 (PMC6491707; doi:10.3389/fimmu.2019.00873)
Supplement: Supplementary file 1 [file Table_1.docx]

|  | **Supplementary Table 1.** | |  |  |  |  |
| --- | --- | --- | --- | --- | --- | --- |
|  | **"Pre and Post-Transplanation" ROC curve data** | | | |  |  |
|  |  |  |  |  |  |  |
|  |  | **miR-155-5p** | **miR-155-5p** | **miR-181a-5p** |  |  |
|  | ***PRE-TX*** | **TCMAR** | **SCR** | **SCR** |  |  |
|  | **AUC** | 0.921 | 0.942 | 0.826 |  |  |
|  | **CUT-OFF** | 0.075 | 0.060 | 0.457 |  |  |
|  | **%SENSITIVITY** | 93 | 100 | 80 |  |  |
|  | **%SPECIFICITY** | 82 | 75.3 | 87.7 |  |  |
|  | **%PPV** | 56.6 | 57 | 53 |  |  |
|  | **%NPV** | 100 | 100 | 100 |  |  |
|  |  | **miR-155-5p** | **miR-181a-5p** | **miR-122-5p** | **miR-148a-3p** |  |
|  | ***POST-TX*** | **TCMAR** | **TCMAR** | **TCMAR** | **TCMAR** |  |
|  | **AUC** | 0.94 | 0.953 | 0.978 | 0.686 |  |
|  | **CUT-OFF** | 0.463 | 0.760 | 4.356 | NA |  |
|  | **%SENSITIVITY** | 91 | 90 | 93 | NA |  |
|  | **%SPECIFICITY** | 95 | 95 | 90 | NA |  |
|  | **%PPV** | 95 | 90 | 71 | NA |  |
|  | **%NPV** | 99 | 97 | 98 | NA |  |
|  |  | **miR-155-5p** | **miR-181a-5p** | **miR-122-5p** | **miR-148a-3p** |  |
|  | ***POST-TX*** | **SCR** | **SCR** | **SCR** | **SCR** |  |
|  | **AUC** | 0.977 | 0.949 | 0.921 | 0.592 |  |
|  | **CUT-OFF** | 0.255 | 0.587 | 2.726 | NA |  |
|  | **%SENSITIVITY** | 93.3 | 91 | 84 | NA |  |
|  | **%SPECIFICITY** | 90 | 88 | 80 | NA |  |
|  | **%PPV** | 89 | 91 | 75 | NA |  |
|  | **%NPV** | 100 | 90 | 90 | NA |  |
|  |  |  |  |  |  |  |
|  | *AUC: area under the curve; PPV:positive predictive value; NPV: negative predictive value* | | | | | |
|  | *TCMAR: T cell mediated acute rejection; SCR: subclinical rejection; NA:not applicable* | | | | |  |

| **Supplementary Table 2.** | |  |  |  |  |  |
| --- | --- | --- | --- | --- | --- | --- |
| **Kruskal Wallis analysis of serum aminotransferase levels between non-rejectors, TCMAR and SCR patients** | | | | | | |
|  |  |  |  |  |  |  |
|  | **P<0.05** |  |  |  |  |  |
| **1st WEEK** |  |  |  |  |  |  |
| AST | 0.067 |  |  |  |  |  |
| ALT | 0.584 |  |  |  |  |  |
| GGT | 0.151 |  |  |  |  |  |
| **Day 15th** |  |  |  |  |  |  |
| AST | 0.724 |  |  |  |  |  |
| ALT | 0.644 |  |  |  |  |  |
| GGT | 0.231 |  |  |  |  |  |
| **1st Month** |  |  |  |  |  |  |
| AST | 0.192 |  |  |  |  |  |
| ALT | 0.089 |  |  |  |  |  |
| GGT | 0.068 |  |  |  |  |  |
| **2nd Month** |  |  |  |  |  |  |
| AST | 0.903 |  |  |  |  |  |
| ALT | 0.635 |  |  |  |  |  |
| GGT | 0.737 |  |  |  |  |  |
| **3rd Month** |  |  |  |  |  |  |
| AST | 0.596 |  |  |  |  |  |
| ALT | 0.262 |  |  |  |  |  |
| GGT | 0.536 |  |  |  |  |  |
| **6th Month** |  |  |  |  |  |  |
| AST | 0.096 |  |  |  |  |  |
| ALT | 0.173 |  |  |  |  |  |
| GGT | 0.103 |  |  |  |  |  |
| **9th Month** |  |  |  |  |  |  |
| AST | 0.180 |  |  |  |  |  |
| ALT | 0.181 |  |  |  |  |  |
| GGT | 0.180 |  |  |  |  |  |
| **12th Month** |  |  |  |  |  |  |
| AST | 0.667 |  |  |  |  |  |
| ALT | 0.304 |  |  |  |  |  |
| GGT | 0.213 |  |  |  |  |  |
| Kruskal Wallis test. Groups: Free clinical event; TCMAR and SCR | | |  |  |  |  |
| A value of P≤0.05 was considered significant. | | |  |  |  |  |
